# Gastrointestinal Motility Disorders and Their Clinical Implications in Cirrhosis

**DOI:** 10.1155/2017/8270310

**Published:** 2017-05-11

**Authors:** Eleni Theocharidou, Ameet Dhar, David Patch

**Affiliations:** ^1^The Royal Free Sheila Sherlock Liver Centre, Royal Free Hospital, Royal Free Hampstead NHS Trust, University College London, London, UK; ^2^Section of Hepatology, Department of Medicine, St Mary's Hospital, Imperial College London, London, UK

## Abstract

Gastrointestinal motility is impaired in a substantial proportion of patients with cirrhosis. Cirrhosis-related autonomic neuropathy, increased nitric oxide production, and gut hormonal changes have been implicated. Oesophageal dysmotility has been associated with increased frequency of abnormal gastro-oesophageal reflux. Impaired gastric emptying and accommodation may result in early satiety and may have an impact on the nutritional status of these patients. Small intestinal dysmotility might be implicated in small intestinal bacterial overgrowth and increased bacterial translocation. The latter has been implicated in the pathophysiology of hepatic encephalopathy and spontaneous bacterial peritonitis. Enhanced colonic motility is usually associated with the use of lactulose. Pharmacological interventions aiming to alter gastrointestinal motility in cirrhosis could potentially have a beneficial effect reducing the risk of hepatic decompensation and improving prognosis.

## 1. Introduction

Disorders of gastrointestinal (GI) motility have been described in patients with cirrhosis and can affect all parts of the GI tract. Cirrhosis has been associated with autonomic neuropathy, increased nitric oxide (NO) production, and gut hormonal changes that can have an impact on GI motility. GI motility disorders are rarely investigated in this population in daily clinical practice. Their clinical implications, however, can be potentially deleterious for patients' nutritional status and risk of infectious complications and hepatic decompensation. These implications are often underestimated but might be at least partially reversible with interventions that aim to alter GI motility, a hypothesis that should be further tested in the future. The aim of this review is to provide an overview of the available evidence on GI motility disorders in cirrhosis and their clinical implications.

## 2. Oesophageal Motility

A small number of studies have addressed disorders of oesophageal motility in patients with cirrhosis. A study that assessed 78 cirrhotic patients without oesophageal varices (EV) using oesophageal manometry and simultaneous 24-hour pH study reported increased frequency of abnormal reflux episodes (55%) and reflux oesophagitis (37%) compared to 30 healthy controls [1]. The lower oesophageal sphincter (LES) pressure was lower in patients with cirrhosis and correlated inversely with the Child-Pugh score. The peristaltic amplitude, duration, and velocity were also abnormal in the same group. Increased NO levels in the systemic circulation of patients with cirrhosis may account for the hypotonic LES leading to increased gastro-oesophageal reflux (GOR).

The presence of EV may further aggravate the abnormalities in oesophageal motility. A study that included 45 patients with EV and 45 healthy controls showed decreased peristaltic wave amplitude and increased frequency of tertiary contractions in the former group, but these abnormalities were not associated with symptoms of GI dysmotility [2]. A manometric study in 45 patients with cirrhosis and EV, 45 patients with cirrhosis without EV, and 20 healthy controls demonstrated decreased peristaltic wave amplitude in the lower oesophagus, increased peristaltic duration, and increased peristaltic velocity [3]. There was no difference in LES pressure and duration of LES relaxation between cirrhotic patients and controls. Although the presence of EV is associated with more pronounced oesophageal motility disorders, the clinical significance of this observation is unclear as these disorders are rarely associated with dysphagia or retrosternal discomfort.

Endoscopic treatment for EV, in particular endoscopic injection sclerotherapy (EIS), has a well-known effect on oesophageal structure and motility. EIS has an impact primarily on the function of the lower oesophagus resulting in decreased amplitude and/or velocity and increased duration of the peristaltic contractions, which may be replaced by nonpropagating contractions [4]. Acid clearance may be prolonged, but this is usually transient and resolves within a week. A short course of antacid therapy is, therefore, justified following sclerotherapy. EIS usually spares the LES. A small study, however, that assessed oesophageal motility prior to and post EIS, showed decreased LES pressure in the latter, but without significant increase in GOR episodes [5]. The mechanism of motility disorders remains unclear, although sclerosant-induced vagal nerve injury has been hypothesized [6]. EIS has been also associated with structural abnormalities, such as sclerosant-induced oesophageal ulcerations and fibrotic strictures [7], and less commonly with oesophageal perforation [8]. Strictures can be associated with dysphagia and may require endoscopic dilatation.

Endoscopic variceal ligation (EVL) has largely replaced EIS on the grounds of comparable efficacy in controlling acute variceal bleeding and eradicating EV and considerably fewer complications. EVL does not seem to have a significant effect on oesophageal motility [4]. Sixty patients were randomized to EIS or EVL following an episode of variceal bleeding. Oesophageal function was assessed by means of oesophageal manometry and 24-hour pH study at presentation and a month later [9]. EIS was associated with decreased peristaltic wave amplitude, increased simultaneous contractions, and increased exposure to pH < 4. No oesophageal dysfunction was observed in the EVL group. Another study showed that EVL partially improved oesophageal dysmotility in 45 patients with cirrhosis at 4–6 weeks following variceal obliteration [2]. The amplitude of the peristaltic wave improved at 5 cm above LES but remained decreased at 10 cm above LES, and the frequency of tertiary contractions remained increased following EVL.

In summary, there is evidence that cirrhosis with or without EV is associated with oesophageal dysmotility, but the clinical significance of this observation has not been studied extensively. Increased incidence of GOR and reflux oesophagitis have been reported in cirrhosis, but symptoms of GI dysmotility, such as dysphagia and chest pain, are uncommonly described in this population, with the exception of patients who develop strictures following endoscopic therapy for EV. EIS is rarely applied, and the sequelae on esophageal function are very rarely encountered. EVL is very effective and safer endoscopic therapy of EV that has replaced EIS.

## 3. Gastric Motility

Nonspecific upper GI symptoms are common in patients with cirrhosis and may relate to impaired gastric emptying. Gastric motility has been studied in patients with cirrhosis using gastric emptying scintigraphy (*n* = 304) [10–18], ultrasonography (*n* = 67) [19–22], electrogastrography (EGG) (*n* = 57) [23–25], and breath tests (*n* = 82) [26, 27]. Prolonged gastric emptying has been demonstrated in 24–95% of patients with cirrhosis and upper GI symptoms that are not attributable to organic GI disorders [10–12]. Most studies excluded patients with diabetes, and there are no studies in patients with nonalcoholic fatty liver disease, in which insulin resistance is a common feature and may account for this disorder. Gastric dysmotility is probably more common in patients with more advanced liver disease [12], although this was not confirmed in all studies, likely due to the small sample size. A strong positive association has been found between delayed gastric emptying and autonomic neuropathy as assessed by standardized cardiovascular autonomic tests [11, 12]. Autonomic neuropathy, including sympathetic upregulation and parasympathetic downregulation, has been described in 30–70% of patients with cirrhosis irrespective of the aetiology [28, 29] and may account for gastric and intestinal dysmotility in these patients. Cisapride, a serotonin 5-HT4 agonist that stimulates the release of acetylcholine from the myenteric plexus, improves gastric emptying in patients with cirrhosis and autonomic neuropathy [12, 21]. An alternative mechanism involves abnormalities in peptic hormones that regulate gastric motility, insulin resistance in cirrhosis that may lead to postprandial hyperglycaemia, high insulin and low ghrelin levels, and abnormalities that are associated with delayed gastric emptying [10]. Increased secretin levels have been also implicated [25]. A third mechanism implicates decreased postprandial portal blood flow due to high resistance in the portal system, which results in stasis and congestion in the gastric wall leading to impaired antral compliance and motility [24].

Apart from gastric emptying, which mainly reflects antral motility, a small study using gastric barostat showed increased gastric accommodation in patients with cirrhosis [30]. Another study, however, that measured proximal gastric size by ultrasonography following a semisolid and liquid meal, showed decreased gastric accommodation in patients with cirrhosis [31]. These results need to be confirmed in further studies.

Although large volume ascites may cause symptoms of early satiety, it does not seem to have an effect on gastric emptying. Two studies that assessed gastric emptying before and after large volume paracentesis found no improvement in gastric emptying following paracentesis, despite symptomatic improvement of early satiety [14, 27].

Finally, a small study in 24 patients with cirrhosis demonstrated that gastric emptying not only is impaired but also is not coordinated with gallbladder contractility, which may account for gallstone formation in these patients [20].

Gastric dysmotility is not without clinical implications in patients with cirrhosis. Abnormalities in gastric motility may account for upper GI symptoms frequently encountered in cirrhosis and might contribute to decreased food intake and poor nutrition in a proportion of patients. Antral dysmotility may have a role in the pathogenesis of GAVE by inducing mucosal mechanical trauma [22]. GAVE, although not specific to cirrhosis, is a well-known cause of chronic blood loss and anaemia in cirrhosis. No correlation seems to exist between impaired gastric emptying and development of hepatic encephalopathy [32].

## 4. Small Intestinal Motility

Prolonged orocecal transit time (OCTT) is a consistent observation in motility studies in patients with cirrhosis. The majority of these studies used lactulose hydrogen breath test to assess OCTT (*n* = 386) [33–42]. One study used radiopaque markers to measure motility in different parts of the GI tract (*n* = 42) [10]. Three studies investigated small intestinal motility, in particular motility of the proximal small intestine, by recording migrating motor complex (MMC) activity using manometric catheters inserted into the proximal small intestine (*n* = 74) [43–45]. A single study used a wireless motility capsule (SmartPill) (*n* = 20) [46]. The most common documented abnormality was prolonged phase 2 occupied by multiple-clustered contractions. Most studies, likely underpowered, failed to show an association of intestinal dysmotility with the severity of liver disease, with only few showing more prominent abnormalities in patients with Child-Pugh C cirrhosis [44, 46]. The single study that assessed small bowel transit time using the SmartPill in 10 patients with compensated and 10 patients with decompensated cirrhosis found more prolonged transit in the latter and showed an association between transit time and the severity of liver disease as reflected in the Child-Pugh score [46]. One study in patients with cirrhosis with and without portal hypertension found motility disorders only in those with portal hypertension; however, the number of included patients was small (12 patients in each group) [36].

Initial studies suggested impaired OCTT only in patients with alcohol-related cirrhosis compared to those with nonalcoholic cirrhosis and healthy controls [47], a finding that had been attributed to underlying alcoholic neuropathy, as intestinal dysmotility has been shown in chronic alcoholics even in the absence of cirrhosis [48]. Further studies showed similar abnormalities in patients with cirrhosis of other aetiologies [40], such as chronic hepatitis B [34] and primary biliary cirrhosis (PBC) [45]. Noncirrhotic patients with chronic hepatitis B had similar OCTT to healthy controls [34]. The latter study found no difference in proximal intestinal dysmotility between PBC patients and those with cirrhosis of other aetiologies [45]. Furthermore, a study in patients with hepatocellular carcinoma (HCC) found that OCTT was significantly more prolonged compared to cirrhotics without HCC [35]. These results suggest that intestinal dysmotility is a feature of cirrhosis irrespective of aetiology, although certain liver diseases, such as alcohol-related liver disease [41], might be associated with higher frequency of motility abnormalities. It is interesting that intestinal dysmotility resolved within six months following liver transplantation in two patients with cirrhosis [49].

The pathogenesis of motility disorders in cirrhosis is not clear, but autonomic neuropathy, NO, and hormonal abnormalities have all been implicated. A study in 48 patients with cirrhosis demonstrated that autonomic neuropathy was more common in patients with delayed OCTT, compared to those with normal OCTT [39]. Autonomic neuropathy was the only independent predictor of prolonged OCTT. However, another study in 32 patients with nonalcoholic cirrhosis showed no association between OCTT and parameters of autonomic function [40]. The exclusion of patients with alcohol-related cirrhosis, in which autonomic dysfunction is probably more prominent, may account for the lack of association in the latter study. Cisapride, increasing acetylcholine release, has been found to shorten intestinal transit time in experimental models of cirrhosis (rats) [50, 51].

A second mechanism involves NO, which is increased in the systemic circulation of patients with cirrhosis, and may account for impaired intestinal motility. NO is an inhibitory neurotransmitter affecting GI smooth muscle contraction. A study in cirrhotic rats demonstrated abnormal intestinal motility in rats with increased NO levels but normal motility in rats treated with a NO synthase inhibitor and normal NO levels [52].

Metabolic and hormonal abnormalities may also account for abnormal small intestinal motility in cirrhosis. Increased postprandial glucose and insulin levels as a result of insulin resistance and decreased postprandial ghrelin levels have been demonstrated in patients with cirrhosis and were found to correlate with prolonged small intestinal transit [10]. Hyperglycaemia and hyperinsulinaemia delay gastric emptying and small intestinal transit in both diabetic and nondiabetic patients [53], whereas postprandial ghrelin levels correlate inversely with glucose and insulin levels. It can be hypothesized that treatment of insulin resistance in cirrhosis may improve intestinal motility in these patients. More studies are required to elucidate the role of these disorders in intestinal dysmotility in cirrhosis.

The most important implication of prolonged small intestinal transit is the potential predisposition to small intestinal bacterial overgrowth (SIBO) and bacterial translocation [54]. The propulsion of intestinal contents by intestinal peristalsis prevents the overgrowth of bacteria. An association has been demonstrated between prolonged small intestinal transit as assessed using the SmartPill and SIBO confirmed by a positive lactulose breath test [55]. Treatment with cisapride improved OCTT and abolished SIBO and bacterial translocation in cirrhotic rats [51]. These observations were confirmed in 12 patients with cirrhosis, in which long-term treatment with antibiotics improved OCTT and abolished SIBO [38]. SIBO can have significant implications in cirrhosis: Firstly, SIBO may account for some common GI symptoms in patients with cirrhosis such as abdominal pain, bloating, and diarrhea. Secondly, it can be associated with malabsorption and nutritional deficits. Thirdly, it may precipitate the development of complications of liver disease via increased bacterial translocation, such as hepatic encephalopathy (HE) and spontaneous bacterial peritonitis (SBP). A study in 45 patients with cirrhosis demonstrated a weak correlation between prolonged small intestinal transit and symptoms of abdominal pain and diarrhea [10]. Although diarrhea is usually associated with accelerated bowel transit, SIBO may explain diarrhea in this setting. Importantly, SIBO may contribute to nutritional deterioration and weight loss in patients with cirrhosis.

SIBO has been associated with SBP, and selective intestinal decontamination with antibiotics prevents recurrent SBP [56]. SIBO was assessed using glucose hydrogen breath test, and small intestinal motility by MMC activity recording, in 20 patients with history of SBP and 20 without history of SBP [33]. SIBO and MMC abnormalities were more common in patients with history of SBP, suggesting that intestinal dysmotility may result in SIBO leading to increased bacterial translocation and episodes of SBP.

A similar pathophysiological mechanism may also apply to the development of HE. A study in 10 patients with alcohol-related cirrhosis and HE, and 18 without HE, demonstrated prolonged OCTT in the first group [57]. An even larger study included 102 patients with cirrhosis, 57 of which had minimal hepatic encephalopathy (MHE) diagnosed with psychometric tests [37]: SIBO was more common in patients with MHE compared to those without MHE, and prolonged OCTT was more common in patients with SIBO compared to those without SIBO. SIBO was the only independent predictor of MHE in multivariate analysis. These results provide the rationale for prevention of complications, such as HE and SBP, with nonabsorbable antibiotics. If it is hypothesized that SIBO is the result of impaired intestinal motility, treatment with prokinetic agents might in theory reduce SIBO by improving motility, obviating the need for intestinal decontamination and alleviating the associated risk of bacterial resistance to antibiotics. This hypothesis and the potential utility of prokinetic pharmacological agents should be tested in future studies.

## 5. Colonic Motility

Data on colonic motility in patients with cirrhosis is scarce and can be confounded by the use of lactulose. Two small studies using radiopaque markers showed accelerated colonic transit (*n* = 25), in particular in the descending colon [58, 59], whereas a third study showed no difference in colonic transit in patients with cirrhosis compared to controls [41]. In one of these studies, accelerated colonic transit was demonstrated in the left colon in patients with decompensated cirrhosis and was associated with diarrhea [58]. In another study, colonic transit time correlated inversely with albumin levels [59]. The study that assessed GI motility using the SmartPill (*n* = 20) showed no difference in colonic transit time between patients with compensated and decompensated liver disease [46]. In summary, there is some preliminary evidence to suggest accelerated left colonic transit in cirrhosis that is more pronounced in patients with more advanced liver disease, but data are derived from very small studies and should be confirmed in larger cohorts. In addition, patients with cirrhosis complicated by HE are often treated with agents that accelerate colonic transit, with lactulose being the most commonly used, which should be taken into account when studying colonic motility in these patients.

## 6. Conclusion

GI motility disorders are not uncommon in patients with cirrhosis and in particular in those with more advanced liver disease and portal hypertension. Oesophageal dysmotility may account for GOR episodes and reflux oesophagitis in this population. Endoscopic therapy for EV was a contributor to oesophageal functional and structural abnormalities in the past but is no longer a concern with the implementation of EVL. Prolonged gastric emptying and impaired gastric accommodation can be associated with poor oral intake and nutrition and may be implicated in the development of GAVE. Small intestinal dysmotility is a consistent finding in cirrhosis and might be implicated in SIBO and increased bacterial translocation. There is some evidence that left-sided colonic motility is accelerated in cirrhosis, but this is not a consistent finding. GI motility disorders may occur more commonly with more advanced/decompensated liver disease. A potential role of GI dysmotility in the risk of hepatic decompensation, and in particular HE and SBP, is often underestimated. It would be very interesting to assess whether pharmacological agents that alter GI motility have an effect on the prognosis of these patients. A potential beneficial effect of beta-blockers and terlipressin, agents that are commonly used in cirrhosis and lower the pressures in the portal venous system, should be also addressed. Finally, there is very limited evidence to suggest that GI dysmotility resolves following liver transplantation, and this could be an area of future research.
